# 

**Published:** 1897-04-24

**Authors:** 


### EARLY EDITION.

**Notices of Births, Deaths, and Marriages** are inserted in this column at a charge of 2s. 6d. for an announcement not exceeding four lines.

### NOTICE TO CORRESPONDENTS.

All MS., Letters, Books for Review, and other matters intended for the Editor should be addressed The Editor, "The Hospital," The Hospital Building, 28 & 29, Southampton Street, Strand, W.C.
The Editor cannot undertake to return rejected MS., even when accompanied by stamped directed envelope.

**Notice to Advertisers and Subscribers.**

All Advertisements, Orders for Copies of The Hospital, and Business Communications should be addressed to **THE MANAGER, (Not to The Editor),** The Hospital, The Hospital Building, 28 & 29, Southampton Street, Strand, London, W.C.
The Cost of Subscription, post free, is as follows:—Per week, 2½d.; per quarter, 2s. 8d.; per half-year, 5s. 3d.; per annum, 10s. 6d. Foreign:—Per annum, 15s. 2d.; payable, in all cases, in advance.

**NOTICE.—Vol. XXI. of "The Hospital" (October, 1896, to March, 1897), in handsome cloth binding, is on sale at the Office of this Journal, price 7s. 6d. Cases for binding the Numbers contained in this Volume, 1s. 6d. Index to Vol. XXI., 2½d., post free.**
**The Monthly Part for May will be ready on May 1st. Price 6d., by post 8d.**

### SCRAPS AND GLEANINGS.

The Queen has sent a subscription of £10 to the Royal Albert Orphan Asylum, Bagshot.

Mr. W. West Linington, F.R.C.S.Eng., has been appointed resident casualty officer at the General Infirmary, Leeds.

Baroness de Hirsch has given a donation of £200 towards the extension and improvement fund of Queen Charlotte's Lying-in Hospital.

The Worshipful Company of Skinners have sent a donation of 10 guineas to the Royal Hospital for Diseases of the Chest, City Road.

The ladies of the Birkenhead branch of the Brabazon Employment Society have sent a most acceptable gift of couch lounges, armchairs, and footstools for the patients in the Birkenhead Infirmary.

The Royal Free Hospital, Gray's Inn Road, has a zealous and hard-working man of affairs in its Chairman of Committee, Mr. Justice Bruce. Sir Gainsford only finished his heavy duties at the Leeds Assizes late on Tuesday afternoon, the 23rd ult., and on the following day, Wednesday, he was punctual in his attendance at the meeting of the weekly board, which is held at the hospital every week. Mr. Cock, Q.C., was also among those present at the meeting.

Annual meetings are very numerous at the present time. From the reports submitted we extract the following statistics: At the West Cornwall Infirmary, Penzance, 148 in-patients were treated; the income amounted to £770, and the expenditure to £843. At the Ripon Dispensary and Cottage Hospital, 50 in and 786 out-patients were treated; the receipts amounted to £422, and the expenses to £408. The patients treated at the Birmingham Children's Hospital in 1896 were 922 in and 12,987 out: an increase on 1895. At the Ilkeston Hospital the patients numbered 89 in and 247 out-patients, at the Newcastle Hospital for Sick Children, 637 and 67,954; at the Bath Royal United Hospital, 1,348 and 9,698; at the Dawlish Cottage Hospital, 57 and 210 respectively. At the Ottery St. Mary Cottage Hospital 58 in-patients were treated.

### The Student of Medicine.

### APPOINTMENTS.

F. W. Abbott, M.R.C.S.Eng., L.R.C.P.Lond., appointed Medical Officer for the Tooting Workhouse of the Wandsworth and Clapham Union. C. R. Box, B.Sc., M.D., B.S.Lond. M.R.C.P.Lond., F.R.C.S.Eng., appointed Resident Assistant Physician to St. Thomas's Hospital. E. A. Burgess, M.R.C.S. Eng., L.S.A., appointed Medical Officer for the Thorncombe District of the Beaminster Union. Dr. Cox, appointed Medical Officer for the Knighton District of the Knighton Union. S. Craddock, M.R.C.S.Eng., L.S.A., appointed Medical Officer of the Cottage Home Schools of the Bath Union. H. Frere, M.B., C.M.Edin., appointed Medical Officer for the Stradbroke District of the Hoxne Union. W. A. Gostling, M.D., M.R.C.S., appointed Medical Officer to the Worthing Infirmary. G. Home, M.D., appointed Clinical Assistant to Out-patients at the Chelsea Hospital for Women, Fulham Road. J. Lockwood, M.R.C.S.Eng., appointed Medical Officer of Health to the Whitley Urban District Council. C. W. Low, M.B., D.P.H., appointed Medical Officer of Health to the Stowmarket Urban District Council. W. A. McCutchan, L.R.C.P. and L.R.C.S.Edin., appointed Assistant Medical Officer to the County and City Asylum, Hereford. S. C. Phillips, M.R.C.S.Eng., L.R.C.P.Lond., appointed Medical Officer for the North Peckham District of the Camberwell Parish. J. H. Reynolds, M.B., C.M.Edin., appointed Special Non-resident Clinical Clerk to the Lock Wards, Royal Infirmary, Edinburgh. N. H. Runciman, L.R.C.P., L.R.C.S.Edin., L.F.P.S.Glasg., appointed Interim Physician to the South Infirmary, Cork. H. Smalley, M.D.Durh., L.R.C.P.Lond., M.R.C.S., Medical Officer of Parkhurst Convict Prison, appointed Medical Officer of Local and Convict Prisons. C. H. Taylor, M.B.Lond., M.R.C.S., &c., appointed Honorary Surgeon to the Derbyshire Royal Infirmary. F. C. Tothill, M.B., C.M.Edin., appointed Medical Officer for the Ashford District of the Staines Union. N. Townsend, L.R.C.P., L.R.C.S.I., appointed Extern Physician to the South Infirmary, Cork. W. Wranham, M.B.Lond., M.R.C.S., L.R.C.P., appointed Assistant House Surgeon at Leicester Infirmary. J. Grant Andrew and T. W. Jenkins have been appointed Assistant Surgeons to the Victoria Infirmary, Glasgow.

**"THE HOSPITAL," Vol. XXI.**

**(October, 1896, to March, 1897),**

